# Supplementary figures and images for: Surface Wettability Modification of Cyclic Olefin Polymer by Direct Femtosecond Laser Irradiation
Source: Nanomaterials (Basel). 2015 Aug 28;5(3):1442–53. doi: 10.3390/nano5031442 (PMC5304633; doi:10.3390/nano5031442)

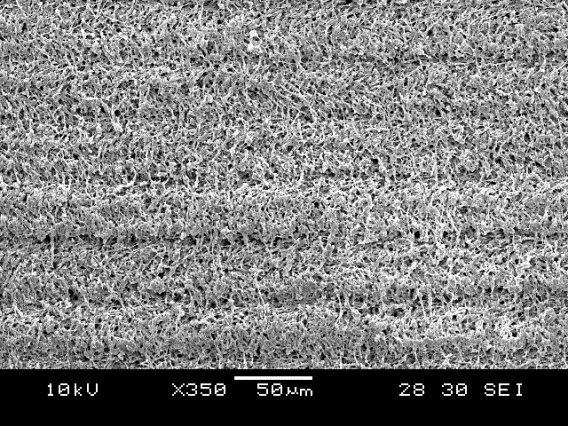

Supplement: Supplementary file 1 [file nanomaterials-05-01442-s001.zip › Lam_Supplementary/SEM/PDR 0.7.png]

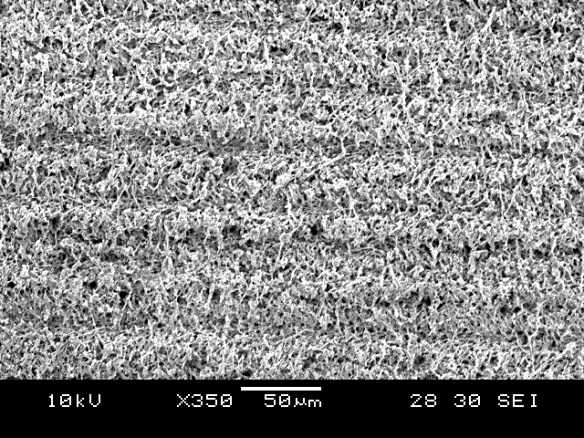

Supplement: Supplementary file 1 [file nanomaterials-05-01442-s001.zip › Lam_Supplementary/SEM/PDR 0.93.png]

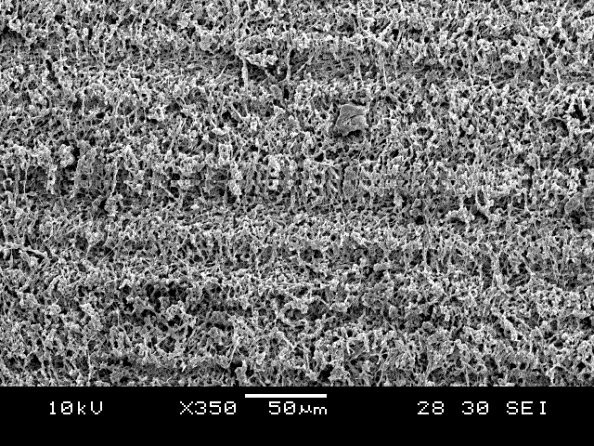

Supplement: Supplementary file 1 [file nanomaterials-05-01442-s001.zip › Lam_Supplementary/SEM/PDR 1.39.png]

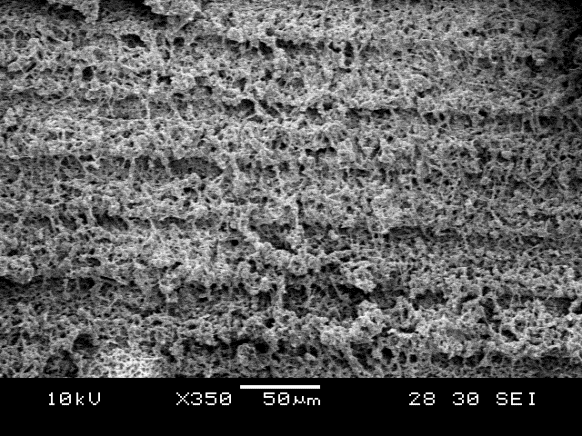

Supplement: Supplementary file 1 [file nanomaterials-05-01442-s001.zip › Lam_Supplementary/SEM/PDR 2.79.png]

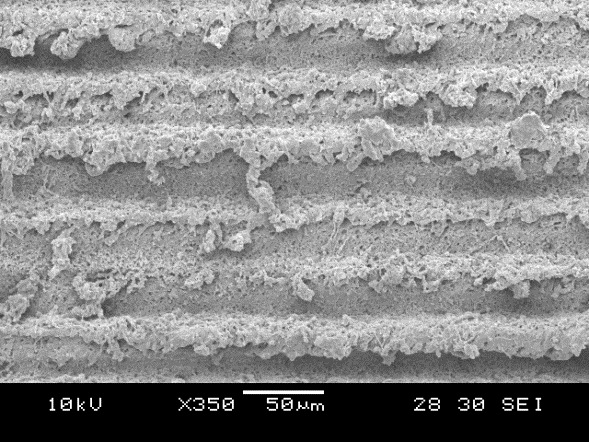

Supplement: Supplementary file 1 [file nanomaterials-05-01442-s001.zip › Lam_Supplementary/SEM/PDR 5.57.png]

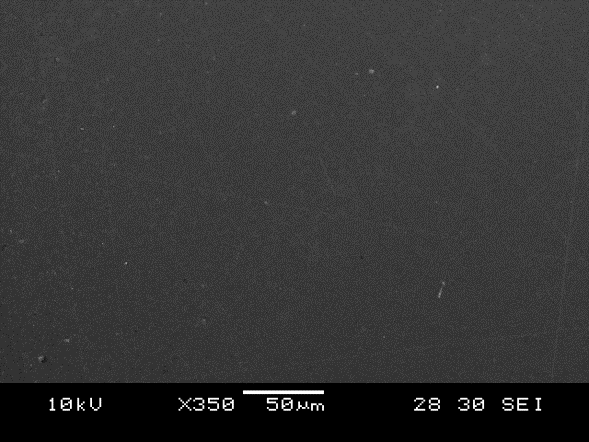

Supplement: Supplementary file 1 [file nanomaterials-05-01442-s001.zip › Lam_Supplementary/SEM/Pristine.png]

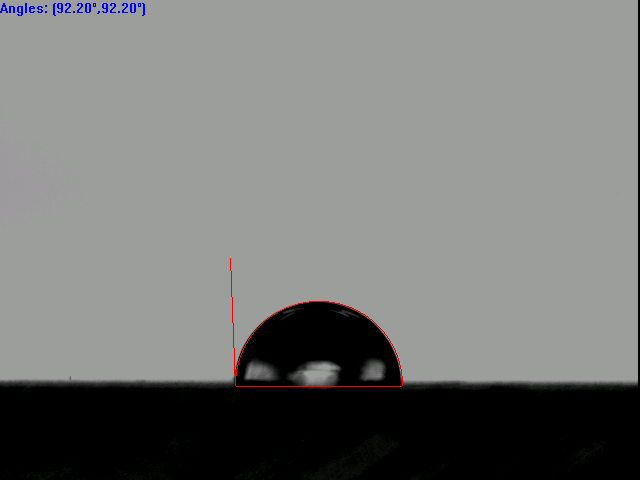

Supplement: Supplementary file 1 [file nanomaterials-05-01442-s001.zip › Lam_Supplementary/WCA/original wet angle.bmp]

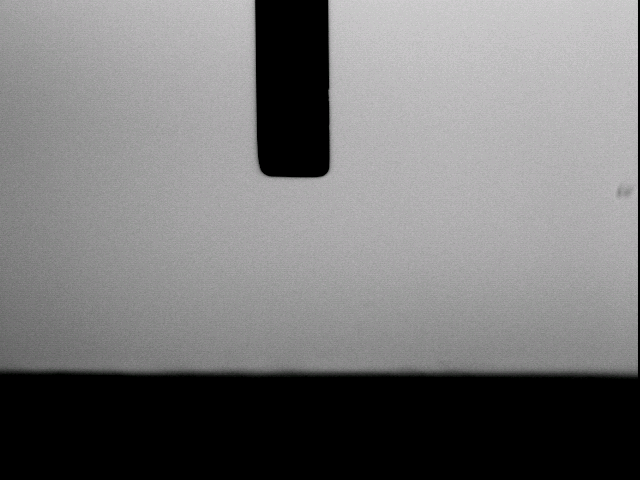

Supplement: Supplementary file 1 [file nanomaterials-05-01442-s001.zip › Lam_Supplementary/WCA/super hydrophilic PDR 0.62 fluence 17.6.bmp]

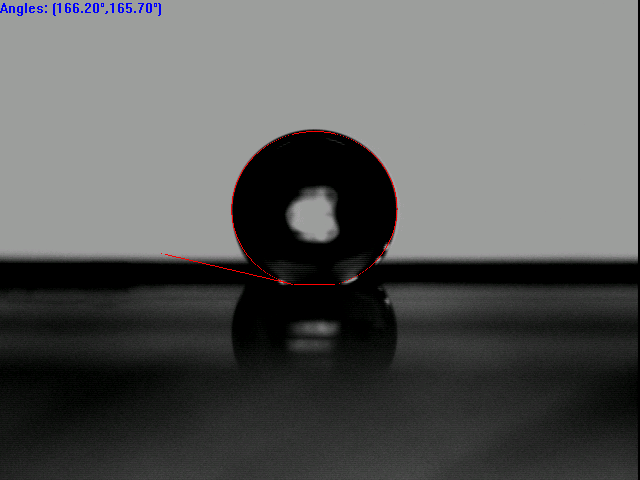

Supplement: Supplementary file 1 [file nanomaterials-05-01442-s001.zip › Lam_Supplementary/WCA/super hydrophobic PDR 6.5 fluence 30.79.bmp]
